# Applicability of the 5S management method for quality improvement in health-care facilities: a review

**DOI:** 10.1186/s41182-016-0022-9

**Published:** 2016-07-19

**Authors:** Shogo Kanamori, Akira Shibanuma, Masamine Jimba

**Affiliations:** Department of Community and Global Health, Graduate School of Medicine, The University of Tokyo, 7-3-1 Hongo, Bunkyo-ku, Tokyo, 113-0033 Japan; IC Net Limited, Land Axis Tower, 27th Floor, 11-2 Shintoshin, Chuo-ku Saitama-shi, Saitama, 330-6027 Japan

**Keywords:** 5S, Lean, Health-care, Quality improvement, Low- and middle-income countries

## Abstract

**Background:**

The 5S management method (where 5S stands for sort, set in order, shine, standardize, and sustain) was originally implemented by manufacturing enterprises in Japan. It was then introduced to the manufacturing sector in the West and eventually applied to the health sector for organizing and standardizing the workplace. 5S has recently received attention as a potential solution for improving government health-care services in low- and middle-income countries. We conducted a narrative literature review to explore its applicability to health-care facilities globally, with a focus on three aspects: (a) the context of its application, (b) its impacts, and (c) its adoption as part of government initiatives.

**Methods:**

To identify relevant research articles, we researched public health databases in English, including CINAHL, PubMed, ScienceDirect, and Web of Science. We found 15 of the 114 articles obtained from the search results to be relevant for full-text analysis of the context and impacts of the 5S application. To identify additional information particularly on its adoption as part of government initiatives, we also examined other types of resources including reference books, reports, didactic materials, government documents, and websites.

**Results:**

The 15 empirical studies highlighted its application in primary health-care facilities and a wide range of hospital areas in Brazil, India, Jordan, Senegal, Sri Lanka, Tanzania, the UK, and the USA. The review also found that 5S was considered to be the starting point for health-care quality improvement. Ten studies presented its impacts on quality improvements; the changes resulting from the 5S application were classified into the three dimensions of safety, efficiency, and patient-centeredness. Furthermore, 5S was adopted as part of government quality improvement strategies in India, Senegal, Sri Lanka, and Tanzania.

**Conclusions:**

5S could be applied to health-care facilities regardless of locations. It could be not only a tool for health workers and facility managers but also a strategic option for policymakers. They could consider 5S as the starting point of a government-led quality improvement initiative for improving safety, efficiency, or patient-centeredness aspects particularly in low- and middle-income countries. However, the evidence base, particularly in resource-poor settings, must be expanded.

## Background

The 5S management method—where 5S stands for the five Japanese words *Seiri*, *Seiton*, *Seiso*, *Seiketsu*, and *Shitsuke—*has been used in the automotive and other industries. These five words, often translated into English as “sort, set in order, shine, standardize, and sustain,” broadly refer to the discipline of cleanliness in any place [1]. The 5S management method (hereinafter abbreviated as “5S”) is a set of practices that aims to generate productivity improvements by creating and sustaining clean and well-organized workplaces [1–4]. It is often called the *commonsense approach* and regarded as a low-cost and technologically undemanding participatory approach that workers can implement regardless of their technical knowledge [5].

5S was originally implemented by manufacturing enterprises in Japan. During the 1980s, it was introduced to the manufacturing sector in the West as the secret behind Japanese industrial development. 5S was eventually applied to non-production settings, such as offices, as well [6]. It has also been applied to health-care facilities as a systematic way to organize and standardize the workplace [7]. In Japan, 5S has been commonly practised at hospitals [8–11]. It has also been recognized as a method for health-care quality improvement in several books published in the USA [7, 12–17].

In the context of the health-care quality improvement, 5S has often been regarded as one of the “lean” tools [18], where lean refers to a set of approaches for continuous improvement that aim to maximize added value by removing all necessary factors that do not generate value [19]. Lean has been recognized as one of the key quality improvement approaches in health-care [20].

5S has recently received attention from health-care professionals as a potential solution to improve the service quality of resource-poor government health-care facilities in low- and middle-income countries. The Japan International Cooperation Agency (JICA) has adopted 5S as part of its technical cooperation scheme to improve health-care service quality and has assisted several low- and middle-income countries [21, 22].

We conducted this study to review the applicability of 5S in relation to the following aspects: (a) the context of application to improve the quality of health-care services; (b) the impacts of application to health-care facilities; and (c) the adoption of the method as part of government initiatives, particularly in low- and middle-income countries. The paper concludes by presenting the policy implications of the 5S application.

## Methods

**Fig. 1:**
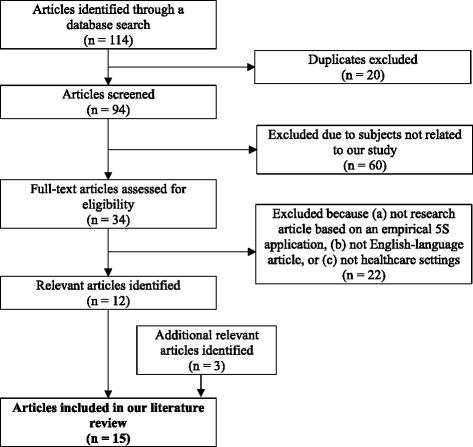
Literature selection flow diagram

We conducted a narrative review of the literature. Since our study was intended to provide an overview of the applicability of 5S from different aspects, rather than answer a clearly defined question, we considered the systematic review inappropriate. Moreover, a systematic review was not suitable because few studies were based on rigorous data collection methods to evaluate the applicability of 5S in health-care facilities (this was our initial assumption, which was confirmed as our literature review progressed). Nevertheless, we referred to the checklist for the Preferred Reporting Items for Systematic Reviews and Meta-Analyses (PRISMA) [23] and, to the extent possible, adhered to the standard methods for the systematic review to identify the relevant literature (Fig. 1).

To identify the relevant empirical studies on the application of 5S in health-care facilities, we investigated the academic literature. We researched public health databases in English, including CINAHL, PubMed, ScienceDirect, and Web of Science, for relevant peer-reviewed research articles published between January 1980 and October 2015 using the keywords “5S,” “five-S,” “lean,” “quality,” “health,” and “hospital.” The search yielded a total of 114 records that contained 94 articles after duplicates were removed. We selected 34 articles for the full-text assessment because the remaining 60 articles were not related to the subjects of our study. We reviewed the main text of the retrieved articles and identified 12 English-language research articles that describe the empirical application of 5S in health-care facilities. We also searched Google Scholar for articles that cite any of these 12 articles (as of October 2015) and identified three additional relevant research articles. We examined the complete texts of the 15 articles to identify the context and impacts of the 5S application; however, we did not assess the validity of the research methods employed in these articles.

To identify additional information pertaining particularly to the context of the 5S application and its adoption as part of government initiatives, we examined the partial or full text of the following: (a) research articles that were among the 34 articles for the full-text assessment but not retained at the final screening stage; (b) research articles, reference books, reports, didactic materials, and government documents that were listed in the reference section of the 15 research articles reviewed in our study; and (c) documents and web-based resources identified through an online search on Google Scholar. To obtain in-depth information about government initiatives mentioned in the research articles, we searched the websites of concerned government agencies for relevant official documents including guidelines, manuals, and reports.

## Results

This study identified several key factors pertaining to the context and impacts of the 5S application. They were the implementation settings, applied tools or methods, changes resulting from the 5S application, and the objective of the intervention in the context of quality improvement (Table 1). Among the 15 empirical studies, the quantitatively measurable changes are presented in the nine articles [24–32]. Changes perceived by health workers are presented in one article, too [33].

**Table 1:** Research articles on the empirical application of 5S to health-care facilities

Author(s)	Settings	Tools/methods applied	Changes resulting from 5S application	Objective in quality improvement context
Al-Araidah et al. [24]	Inpatient pharmacy at a local hospital, Jordan	5S, DMAIC	Potential reductions of more than 45 % were observed in the drug dispensing cycle time	Lean
Chadha et al. [25]	Emergency department at SD Mission Hospital, India	5S visual management, value stream mapping, one-piece flow, standard operating procedures	Improved process flows, increased capacity, and shorter stays for all patient classes were observed	Lean
Farrokhi et al. [26]	Operating room at Virginia Manson Medical Center, USA	5S, value stream, Kaizen event	The number of instruments for minimally invasive spine surgery was reduced by 70 % (from 197 to 58) and setup times decreased by 37 % (13.1–8.2 min, *p* = 0.0015). The potential institutional annual cost savings of US$2.8 million was expected	Lean
Ikuma and Nahmens [27]	Five departments (central supply, histology laboratory, ICU, medical-surgical impatient care unit, and infusion center) at several Ochsner Health System hospitals in southeastern Louisiana, USA	5S	Goals related to compliance with regulations, ergonomics, or safety were achieved in all the five departments	Lean
Pandya et al. [28]	Eighteen government urban health centers in Rajkot municipality, India	5S	Increases in scores of all five “S” (*p* < 0.001) measured by external evaluators based on a 5S audit tool	(Not explicit)
Rutledge et al. [29]	Core laboratory of a tertiary care pediatric facility, USA	5S, visual controls, single piece flow, standard work	The mean turnaround time for creatinine was reduced from 54 to 23 min accompanied by increased testing volume (20 %), monetary savings (four full-time equivalents), decreased variability in turnaround time, and better space utilization (25 %)	Lean
Venkateswaran et al. [30]	Three different hospitals’ central warehouses at Ochsner Health System, USA	5S	Increases in inventory turnover by 30 % in a hybrid 5S (integrated with inventory management techniques and process improvement tools) application site and 4.0 and 43.0 % in two traditional 5S application sites	Lean
Waldhausen et al. [31]	A surgical clinic at Seattle Children’s Hospital, USA	5S, work balance, standard work	Face-to-face provider-patient time increased by 30 to 61 % at 30 days, 58 % at 60 days, and 59 % at 1 year. Satisfaction survey problem scores improved and were sustained	Lean
Withanachchi et al. [32]	Castle Street Hospital for Women in Colombo, Sri Lanka	5S	The infection rate in the post-Caesarean section reduced by 52 % and the stillbirth rate by 33 % over the 2-year period during which 5S was implemented	TQM
Kanamori et al. [33]	A health center in Tambacounda Region, Senegal	5S	Changes perceived by health center staff members, including reduction in time searching for items, improved ability of staff to move around in the office, reduction in waiting time for patients, better directions for patients, and an improved sterilization process	Lean
Esain et al. [35]	Multiple locations delivering acute and community care under the National Health Service, UK	5S	–	Lean
Gabow et al. [36]	Denver Health, USA	5S	–	Lean
Ishijima et al. [37]	46 public hospitals, Tanzania	5S	–	TQM
Patwa et al. [34]	One primary health-care facility and its two sub-centers in Ahmedabad district, India	5S	–	(Not explicit)
Pertence and Melleiro [38]	Sao Paulo University Hospital, Brazil	5S	–	Quality management

### Context of 5S application for quality improvement

Our review identified service areas and geographical locations involved in the empirical 5S application. 5S was applied to primary health-care facilities [28, 33, 34] and different locations or sectors of hospitals, including a pharmacy [24]; an emergency department [25]; an operating room [26]; multiple departments (central supply, histology laboratory, ICU, medical-surgical inpatient care unit, and infusion center) of several hospitals [27]; a laboratory [29]; a surgical clinic [31]; multiple locations of hospitals (or without specific information about target locations) [32, 35–38]; and central warehouses [30]. Of the 15 studies, six were conducted in the USA [26, 27, 29–31], one in the UK [35], and eight in low- and middle-income countries, namely Brazil, India, Jordan, Senegal, Sri Lanka, and Tanzania [24, 25, 28, 32–34, 37, 38].

Depending on the studies, 5S was combined with other tools and its application was meant for different quality improvement goals. Ten of the 15 empirical studies involved the application of 5S only [27, 28, 30, 32–38], whereas the other five studies combined several tools and methods including 5S [24–26, 29, 31]. In addition, 5S was regarded as a method under the framework of lean health-care by authors in ten studies [24–27, 29–31, 33, 35, 36] and toward better quality management (or total quality management (TQM)) in three studies [32, 37, 38].

Several studies presented the perceived roles or stages of the 5S application in the quality improvement context. 5S was considered to serve as an initial step toward TQM [32], as a foundation for continuous improvement [35], as a foundation for the lean tools to establish a self-ordering, self-regulating environment of sustainable change [36], and as a solution to improve the disorderly work environment that serves as a potential bottleneck in providing adequate services [33].

Eight empirical studies focused in low- and middle-income countries, but the resource levels of the studied facilities were not necessarily described in the articles. One of them, based at a health center in Senegal, highlighted the facility’s chronic resource constraints and its extremely disorderly work environment characterized by full of unwanted items kept everywhere unattended before the 5S application [33]. However, in the remaining seven studies, it was not clear whether the health-care facilities faced the typical problems prevalent in those countries, such as financial and human resource constraints.

### Impacts of 5S application to health-care facilities

Ten empirical studies (nine quantitative and one qualitative studies) presented changes resulting from the 5S application and explicitly stated the research methods in the articles reviewed (Table 1). All nine quantitative studies presented measurable changes by comparing the status before and after the interventions without adopting explicit measures to control for potential confounding factors. The qualitative study presented health workers’ views on the changes attributable to the application of 5S in their workplace, daily routines, and services provided. In cases where several tools were utilized in the intervention, it was not possible to identify the extent to which 5S contributed to the changes. One study simply focused on score increases measured for each S (sort, set in order, shine, standardize, and sustain), whereas the remaining nine studies highlighted positive changes in the quality of health-care. Based on the classification of the health-care quality dimensions proposed by the Institute of Medicine (USA) [39], these changes included measures pertaining to three areas: (a) efficiency, (b) safety, and (c) patient-centeredness.

The efficiency measures included improvements to the work processes, potential cost reductions, and increases in physical space [24–26, 29, 30, 33]. The changes resulting from the 5S application were presented as potential reductions of more than 45 % in the drug-dispensing cycle time [24]; improved process flows, increased capacity, and shorter stay for all patient classes [25]; a 70 % reduction in the number of instruments used in minimally invasive spine surgeries (from 197 to 58) and a 37 % decrease in setup times (13.1–8.2 min, *p* = 0.0015); potential institutional annual cost savings of US$2.8 million [26]; a reduction in the turnaround time for a typical test, an increase in the number of tests, cost savings, reductions in the dispersion of the turnaround time, and better space utilization [29]; increases in inventory turnover by 30 % in a hybrid 5S application site and 4.0 and 43.0 % in two traditional 5S application sites [30]; and a reduction in the time involved in searching for items and an improvement in their ability to move within the office after the introduction of 5S [33].

Safety measures included improved ergonomics resulting from the rearrangement and removal of items to eliminate safety violations and improved compliance with regulations [27], 52 % reduction in the post-Caesarean infection rate and 33 % reduction in the stillbirth rate over the 2-year period [32], and an improved sterilization process [33].

The assessment of patient-centeredness measures was based on the time spent on direct patient care increasing from 30 to 61 % after 30 days and improvements in patient satisfaction [31] and reduction in waiting time for patients and better directional indications for patients [33].

### Adoption of 5S application as part of government initiatives

This review highlighted the application of 5S as part of government initiatives. Of the 15 research articles reviewed, five involved empirical 5S application as part of government initiatives, and these five studies were all concentrated in low- and middle-income countries. In these studies, the 5S application was initiated as part of the local governments’ programs in India [28, 34] and national strategies for health-care quality improvement spearheaded by health ministries in Senegal, Sri Lanka, and Tanzania [32, 33, 37].

Other types of publications presented case studies on the adoption of 5S as national strategies in low- and middle-income countries. The Castle Street Hospital for Women in Sri Lanka is the first documented case of 5S application to a government hospital in a low- or middle-income country [32]. Achievements at the Castle Street Hospital led to a pilot study, conducted between 2005 and 2007, to institutionalize 5S at five government hospitals in Sri Lanka [40]. In 2009, the health ministry of the Sri Lankan government initiated a project with the technical support of JICA to improve the quality and safety of health-care facilities in the whole country (Ministry of Healthcare and Nutrition, Project document: improvement of quality and safety in healthcare institutions in Sri Lanka, unpublished). The implementation of the project resulted in the adoption of 5S as part of the national strategies of the Sri Lankan government’s health ministry [41].

Starting in 2007, 5S was introduced to government hospitals in African countries under the framework of JICA’s Asia Africa Knowledge Co-creation Program (AAKCP). With the aim of applying Sri Lanka’s successful experience to Africa, the program provided assistance in introducing 5S-KAIZEN-TQM to pilot government hospitals, first in eight countries (Eritrea, Kenya, Madagascar, Malawi, Nigeria, Senegal, Tanzania, and Uganda; phase I: 2009–2013) and then in another seven countries (Benin, Burkina Faso, Burundi, the Democratic Republic of Congo, Mali, Morocco, and Niger; phase II: 2009–2013) [22, 42]. It was reported that the pilot introduction of 5S-KAIZEN-TQM in these government hospitals in the 15 African countries led to an improvement in the visual management of the workplace as well as the service delivery process [43]. These pilot initiatives led to the formulation of new technical cooperation projects that included 5S as part of the activity components in several participating countries. Those projects resulted in the adoption of 5S as a mainstream strategy for quality improvement in health-care services in Senegal and Tanzania [44–46].

## Discussions

Our literature review identified several key findings about the applicability of 5S in health-care facilities. It illustrated the empirical application of 5S in primary health-care facilities and a wide range of hospital areas in Brazil, India, Jordan, Senegal, Sri Lanka, Tanzania, the UK, and the USA. This finding, along with the housekeeping nature of 5S [5], indicates that 5S could be applied regardless of the locations of health-care facilities. The review also suggests that 5S, a tool that evolved in high-income countries, could improve the health-care quality even in low- and middle-income countries. In addition, the empirical studies presented impacts of the 5S application on quality improvements in the three dimensions of efficiency, safety, and patient-centeredness. These dimensions could be used as viewpoints to identify expected outputs and indicators when a 5S implementation strategy is designed.

Our review identified the role of 5S as a foundation or starting point for quality improvement. This finding could be supported by several normative descriptions in publications pertaining to the application of 5S in health-care facilities. 5S is described as the foundation for all activities aimed at increasing productivity and flow, improving quality, and reducing costs [7]. 5S is also considered to be the building block or the foundation upon which lean health-care rests [47]. It is also defined as the process that provides the foundation for building a lean health-care environment [14, 15].

This study also highlighted the taxonomic issues surrounding the terms “5S,” “lean,” and “TQM.” Depending on the studies, the application of 5S was meant for different objectives, namely lean health-care or TQM. In their narrative review, Powell et al. [20] classified the quality improvement models into five categories, including TQM and lean thinking, and regarded 5S as part of the lean tools. TQM was also considered as an approach interchangeable with continuous quality improvement (CQI) [20]. In contrast, some empirical studies considered 5S as a step toward TQM [32, 37]. In some context, the 5S approach toward TQM was represented as “5S-KAIZEN-TQM,” which was also interchangeably referred to as “5S-CQI-TQM” [45]. Thus, although 5S is commonly considered as a starting point toward lean health-care or TQM, no consensus has been established on the taxonomy involving 5S, lean, and TQM.

In this study, 5S has appeared as part of government initiatives in low- and middle-income countries since the 2000s. 5S has evolved as a lean tool for health workers and facility managers in high-income countries; however, the review findings indicate that 5S has become a strategic option for policymakers to start a government-led quality improvement initiative in those countries.

Thus, our literature review filled knowledge gaps about the applicability of 5S; nevertheless, it also identified areas that need to be further studied. First, 5S’s low-cost and technically undemanding nature implies its appropriateness in health-care facilities facing resource constraints; however, our review results were not sufficient to support this hypothesis. Second, the empirical studies did not provide sufficient insights into the cost-effectiveness, viable scale-up mechanisms, or sustainability of the application of 5S in the health systems. These could be areas of further studies to understand the applicability of 5S, particularly in low- and middle-income countries. Since our literature review was limited to publications in the English language, the identified articles do not necessarily reflect the actual distribution of the 5S practices globally. Furthermore, publications other than peer-reviewed research articles cannot generally be retrieved and selected in a systematic and unbiased way; consequently, our study might suffer from a publication bias to a certain extent.

## Conclusions

5S could be applied to health-care facilities regardless of locations. It could be not only a tool for health workers and facility managers but also a strategic option for policymakers. They could consider 5S as the starting point of a government-led quality improvement initiative, or more specifically, for improving safety, efficiency, or patient-centeredness aspects, particularly in low- and middle-income countries. The low-cost nature of 5S implies that this method is an appropriate initial step toward quality improvement even among resource-constrained health-care facilities. However, the evidence base on its applicability in such settings is limited, and further research is required in this area. In addition, to understand its applicability in the context of strengthening health systems in low- and middle-income countries, the cost-effectiveness, viable scale-up mechanisms, and sustainability of 5S application also need to be further studied.

## Abbreviations

AAKCP, Asia Africa Knowledge Co-creation Program; CQI, continuous quality improvement; JICA, Japan International Cooperation Agency; TQM, total quality management
